# Value Addition Employing Waste Bio-Materials in Environmental Remedies and Food Sector

**DOI:** 10.3390/metabo13050624

**Published:** 2023-05-01

**Authors:** Akriti Taneja, Ruchi Sharma, Shreya Khetrapal, Avinash Sharma, Rupak Nagraik, Baskar Venkidasamy, Manju Nath Ghate, Shavkatjon Azizov, Somesh Sharma, Deepak Kumar

**Affiliations:** 1 School of Bioengineering and Food Technology, Shoolini University, Himachal Pradesh, Solan 173229, India; 2 Department of Oral and Maxillofacial Surgery, Saveetha Dental College and Hospitals, Saveetha Institute of Medical and Technical Sciences, Saveetha University, Chennai 600077, India; 3 School of Pharmacy, National Forensic Sciences University, Gandhinagar Gujarat 382007, India; 4 Laboratory of Biological Active Macromolecular Systems, Institute of Bioorganic Chemistry, Academy of Sciences Uzbekistan, Tashkent 100015, Uzbekistan; 5 Department of Pharmaceutical Chemistry, Tashkent Pharmaceutical Institute, Tashkent 100015, Uzbekistan; 6 Department of Pharmaceutical Chemistry, School of Pharmaceutical Sciences, Shoolini University, Solan 173229, India

**Keywords:** by-products, food squander, metabolites, reutilisation, sustainability valorisation

## Abstract

Overall, combating food waste necessitates a multifaceted approach that includes education, infrastructure, and policy change. By working together to implement these strategies, we can help reduce the negative impacts of food waste and create a more sustainable and equitable food system. The sustained supply of nutrient-rich agrifood commodities is seriously threatened by inefficiencies caused by agricultural losses, which must be addressed. As per the statistical data given by the Food and Agriculture Organisation (FAO) of the United Nations, nearly 33.33% of the food that is produced for utilization is wasted and frittered away on a global level, which can be estimated as a loss of 1.3 billion metric tons per annum, which includes 30% cereals, 20% dairy products 35% seafood and fish, 45% fruits and vegetables, and 20% of meat. This review summarizes the various types of waste originating from various segments of the food industry, such as fruits and vegetables, dairy, marine, and brewery, also focusing on their potential for developing commercially available value-added products such as bioplastics, bio-fertilizers, food additives, antioxidants, antibiotics, biochar, organic acids, and enzymes. The paramount highlights include food waste valorization, which is a sustainable yet profitable alternative to waste management, and harnessing Machine Learning and Artificial Intelligence technology to minimize food waste. Detail of sustainability and feasibility of food waste-derived metabolic chemical compounds, along with the market outlook and recycling of food wastes, have been elucidated in this review.

## 1. Introduction

Taking into consideration that approximately 690 million people worldwide are impoverished of fundamental food requirements and are undernourished, wasting food is unethical and not just economically but also morally indictable. The COVID-19 pandemic confers Food Service Sector with unprecedented opposition and challenges, threatening the food service enterprises [1]. Stay-at-home measures due to the pandemic and lockdowns led to a change in the behavior of consumer patterns. The growth of the relevancy of digital tools and technologies has also been seen in numerous sectors during the pandemic. In addition to confirming food safety and preventing the spread of the coronavirus, strict sanitation rules and regulations applied to businesses also reduce compliance with maintainable business practices because they have an impact on the shares of food waste and already widely used measures to reduce food waste [2]. Food delivery services further aggravate the usage of packaging using plastics. Value-added processes were reconfigured to adapt to consumer needs and behavior accordingly [3]. Food service providers even partnered with farmers for the collection of food waste and then subsequently used them as animal feedstock or fertilizers. Empirical research was undertaken to check the determinants of industrial symbiosis using 3D technology and their reinforcement; however, food services and farmers have adopted anecdotal evidence of industrial symbiosis. This process has reduced food wastage and has helped build more responsive and pliable food supply chains [2,4]. Approximately 30–55% of the production of vegetables and fruits in the country goes to waste per annum, and electricity consumption of more than 50% results in the environmental burden of food waste treatment [5]. High levels of biochemical oxygen demand, chemical oxygen demand, and other impurities are formed by the waste from the food industry. Although the composition has a detrimental effect on the planet, animal, and human health, it also yields a wide range of goods with additional value while having a lower production cost [6]. Food wastes cause significant environmental problems when they are disposed of, which is why in the past ten years, research has focused on finding ways to use food waste to create products with value-added, energy, and lessen the dangerous environmental properties associated with the disposal of food waste [7]. The majority of business sustainability reports ignore environmental risks and plastic litter. Manufacturers are becoming more responsible for packaging, but companies are hardly making an effort to decrease plastic waste, particularly in areas without adequate waste management infrastructure [8].

## 2. Food Squander/Waste (FW): An Overview

FW is generally produced via food processing plants, kitchens, eateries, restaurants, agricultural markets, and industries. Some parts of spoiled food, plant waste, cooked and uncooked animal waste, and remnant food are included in the food squander. The composition of FW differs demographically, and it is quite heterogeneous, but the main components are proteins, carbohydrates, and lipids. As per the data, the moisture content in food wastes is tentatively around 74–90%, 80–97% (Volatile Solids to Total Solids ratio), and carbon to nitrogen ratio (14.7–36.4) [9]. The residues of FW contain an enormous quantity of nutrients such as potassium, which is requisite for the reproduction and growth of plants; nitrogen, which is concerned with environmental impact; and phosphorus, which is essential for energy transfer and photosynthesis. The five steps of the food supply chain—manufacturing, dispensation, storage, distribution, handling, and market consumption—are where food waste occurs [10]. The amt of food wasted per person per day or per year is known as the FW generation rate. It relies on factors viz. inadequate awareness, impeded transportation, overproduction, consumer behavior, excess storage, and indigent packaging and handling [11]. 

### 2.1. Types of Food Waste

#### 2.1.1. Agricultural Residues

Depending on their availability, agricultural leftovers can be distinguished from other solid fuels viz char briquettes, wood, and charcoal based on certain properties [6] The agricultural residues are divided into process residues and field residues, as depicted in Table 1 and Table 2. Stems, seeds, straw, molasses, roots, shells, peel, stubble, and husks are examples of process wastes that are utilized for animal feed, fertilizers, and soil enhancement. While after the agricultural harvesting process, field leftovers (seed pods, leaves, stems, and stalks) are still visible in the field. Using farm waste in a regulated manner can increase irrigation efficiency and reduce soil erosion. As a natural antibacterial, lemon peels, green walnut husks, and pomegranate peels can be utilized. Waste material generated from organic compounds represents a source for producing mushroom-based products (bio-fertilisers, bio-char, bio-fuels) [12]. 

#### 2.1.2. Industrial Waste

Organic residues and pollutants are released through the food manufacturing industries (chips, confectionery, meat, fruits, vegetables, and juices) such as wheat straw, rice straw, bran, orange peel, banana peel, potato peel, dairy waste, sugarcane bagasse, soybean waste, marine waste, lignin, ash, cellulose, moisture, nitrogen, and hemicellulose, and these constituents can digest biochemically, which can produce various useful products such as bioethanol and biogas [27,28,29]. Oil production in industries, a large amount of residues left after the extraction of oils from seeds; are called oil cakes and they contribute to air and water pollution since they because of oil, grease, fat, deferred solids, and dissolved solids. The aforementioned Argo-industrial residues are cost-effective, including a large number of elements, and have endless potential for use as substitute substrates for the fermentation process [6]. 

## 3. Food Waste Management

Sustainable development targets that “By 2030, to halve per capita global food waste at the retail and consumer levels and reduce food losses along production and supply chains, including post-harvest losses” and a specific target (12.3) was included on the topic. Scientists and governmental organizations have developed and continuously tested a variety of concepts and solutions to address the and managing food waste problems. Black soldier fly (BSF) rearing is a potential sector because of its versatility and variety of uses in fields including food waste management, animal feed, and the creation of bioactive compounds. The effective generation of BSF larvae for digesting food waste and its change to beneficial chemicals is made possible by the Internet of Things (IoT) deployment, which includes sensors/devices, software, and other components [15,30]. Due to their critical function in permanent magnets, lamp phosphors, catalysts, rechargeable batteries, etc., rare-earth elements (REEs) are working in green economy. Because China now generates more than 90% of the world’s output of REEs and has tightened export limitations, the supply of REEs to the rest of the world is in jeopardy. Just 1% of the rare earth utilized in outdated consumer goods is recycled. Lanthanum and cerium, which are more plentiful elements, are produced in excess during the primary mining of REE ores for neodymium. Neodymium recycling can thereby lessen the overall amount of REE ores that need to be mined [16]. Food waste at the trade level can be decreased if products limited shelf life or those that are about to expire are discounted so that customers can buy them before they degrade. Perishable food products should not be kept in excess as a result. These food items could be distributed to food banks or charitable groups for the needy, including the homeless, low-income individuals who cannot afford to feed their families, and the destitute. A variety of bio-based goods can be made using FW as well [31,32]. Food waste management’s economic benefits are determined by the local tipping gate price that the facility charges; however, new income streams from the sale of biofuels, biopower, or bioproducts can minimize this dependency [17]. Producing biofuels and its biochemicals from plants, lignocellulosic wastes, and algae offers importance in terms of greenhouse gas emissions compared to their fossil equivalents, while there are trade-offs for other environmental effects and sustainability considerations [33,34]. The application of modern techniques such as omics, which include metagenomics, metabolomics, wasteomics, transcriptomics, diseaseomics, and proteomics. A lasting solution for loss management and environmental issues may be found in the control and reduction of food waste via the use of artificial intelligence (AI) and enzymatic processes [35].

### 3.1. Metabolomics

In 1999, the metabolomics concept was first proposed by Nicholson [36]. It allows for both qualitative and quantitative investigation and is used to characterize tiny compounds or metabolites (1500 Da), while the range could be higher (30–3000 Da) [37,38]. For this purpose, a variety of high-throughput analytical technologies, including nuclear magnetic resonance (NMR) spectroscopy, liquid and gas chromatography–mass spectrometry (LC-MS and GC-MS), capillary electrophoresis mass spectrometry (CE-MS) are widely used. Studies on metabolism employ either untargeted or targeted approaches [39,40]. Non-targeted metabolomics, which uses detectors such as time-of-flight (TOF), Fourier-transform ion cyclotron resonance (FT-ICR), and Orbitrap, can identify thousands of metabolites and is biased toward high-resolving power, detecting as many metabolites as possible without quantification [40]. Targeted metabolomics, in contrast, uses a triple quadrupole (QQQ) detector to target and quantify a particular group of molecules in order to obtain highly sensitive detection and quantification [41,42,43,44]. 

The most important goals to provide and guarantee the needs of a world population that is expanding faster and faster are food quality, food authenticity, and food safety. Consumers demand far higher levels of security along with better knowledge about the molecular makeup, origin, and authenticity of food, as well as the impact of technological change on both human health and chemical and nutritional content. A significant contribution to the pro-filing of metabolites in complex materials, such as foods and food byproducts, is made by the green (NMR-based metabolomics) foodomics. These products can easily extract bioactive substances like prebiotics or antioxidant metabolites [45]. In the analysis of FW’s metabolomics profile, few studies have been conducted to produce vinegar and wine from pineapple pulp and peel characterized by GC-MS and ultra-high performance liquid chromatography (UHPLC) hyphenated with quadrupole-time-of-flight tandem mass spectrometry (UHPLC-QTOF-MS) [46], essential oils from plants in the genus Lavandula, mainly *L. intermedia* and *L. angustifolia*, functional foods and nutraceuticals from by-products of *Vicia faba* beans, and supplements with therapeutic uses from *Passiflora mollissima* seeds confirmed by using (UHPLC-QTOF-MS) [47,48,49]. Using a different technique, like nuclear magnetic resonance (NMR)-based metabolomics analysis revealed alterations in primary and secondary metabolites (e.g., amino acids, carbohydrates, and phenolics) in banana fruits during postharvest senescence [50]. 

According to a research, the combination of untargeted metabolomics and gold nanoparticle (AuNP)-assisted laser desorption/ionization mass spectrometry imaging (LDI-MSI) provides complementary information about the metabolism, including the relative abundance and spatial distribution of metabolites in various banana pulp variants at various stages of postharvest senescence. A significant effect on the amino acid and monoamine levels was observed. These findings imply that tissue-specific metabolic alterations may be responsible for the formation of quality attributes during postharvest senescence in bananas [51]. Open-source databases, robust prediction models, and marker validation all support the conversion of descriptive data into knowledge [52]. To develop, gather, and publish data in a uniform style for comparison across databases and countries, we targeted food composition databases and specialty databases [53]. Substantial quantities of debris, primarily citrus peel and seeds, are produced annually by the fruit juice industry. Limonin was chosen to be the principal representative component in citrus trash and to be investigated for new biological functions. Zebrafish larvae were used to explore the metabolomic reaction triggered by limonin. Depending on the exposure concentration of limonin, the differential metabolites (DMs) change. According to enrichment analysis, the recently found DMs for limonoids and citrus waste were linked to inflammation and neurologic illnesses, including epilepsy. It was discovered that limonin could correct an amino acid issue so that epilepsy neuroprotection can be taken. Researchers’ discoveries gave citrus waste and limonoids a new bio-function and use [54]. Acetic acid and isovaleric acid were found in a research work to have significant odor activity values (OAVs) in fresh coconut water. The substances in aged coconut water vinegar with the highest OAVs were phenylethyl acetate, isoamyl acetate, and benzaldehyde, which had scents resembling almond, banana, or pear. The vinegar made from coconut water was full of phenylalanine and other important amino acids. Through route analysis, three important metabolic substrates (aspartate, glutamate, and pyruvate), seventeen important metabolic pathways were discovered. According to sensory evaluation, aged vinegar tastes better. Repurposing the savory and nutritious liquid by converting mature coconut water into vinegar is an excellent strategy [55]. Brans and other cereal and oilseed by-products have become very important in food technology because they contain high-quality protein. In this context, microwave-assisted and combination microwave-assisted enzymatic extraction techniques were used to extract plant-based proteins and antioxidant chemicals from sesame bran. The effects of independent variables—process time (10–120 min), enzyme concentrations (0.1–2.40 AU/100 g), and temperature (25–55 °C)—were all discovered to have a significant impact on total phenolic content, protein yield, and antioxidant capacity values using a central composite design and the response surface methodology [56].

### 3.2. Artificial Intelligence and Machine Learning

Food wastage primarily occurs in the supply chain in developing countries and at the consumer end in developed countries [57]. Computerization and technical platforms that enable system within food supply chains can both help with the management of agricultural resources and the decrease in food waste. According to studies, supply chains with state-of-the-art technological platforms can reduce food waste by up to 50%; the difficulty of the supply chain and the perishability of the raw materials might be helpful indicators of the application of technology [58]. Mobile apps such as Fridge Pal, LeftoverSwap, and EatChaFood that distribute leftover food are becoming more popular due to their advantages for sustainability. However, little is known about the chances offered to companies to enter the Bottom of the Pyramid (BoP) sector. This study examines how mobile apps for reducing food waste can assist in the co-creation of sustainable value at the BoP, drawing on the theories of means–end, affordance, and Service-Dominant (S-D) logic. In Sri Lanka, information was gathered using semi-structured interviews using a laddering method. Although respondents’ perceptions of app functionalities are comparable, there are glaring discrepancies in the perceived affordances and end goals, which may make it difficult to co-create value [59,60,61]. Using AI and Machine Learning (ML) solutions that help in managing food waste, scaling up operations, and remaining relevant in a dynamic market environment, the food industry is currently seeing an increase in innovative start-ups and tech company collaborations, as shown in Table 3 [62]. Smart scales, AI meters, and cameras are employed to differentiate between various sorts of Foods and assess the food quality. The technologies, which are created utilizing ML algorithms, assist by eliminating needless FW and decreasing the food waste. Notable technical advancements of the Industry 4.0 era is artificial intelligence (AI), which presents an unmatched possibility to transform the food economy from a linear to a circular model. In addition to being efficient at handling ambiguity and missing data, learning from experience, and addressing poorly defined problems, these methodologies have gained popularity in offering substitute computational methods to address solid waste management (SWM) issues [61,63].

## 4. Significant Wastage in Various Food Processing Segments and Their Valorisation

The vegetable and fruit industries engender an extensive proportion of pollutants and other solid wastes, which consist of organic materials, discarded fruit peels, seeds, skin, stones, etc., whereas wastes contain the liquid portion of wash water and juice [68]. Depending on the fruit, 30–50% of the by-products formed through fruit and vegetable processing are used to produce biogas, compost, and fertilizer through the manufacturing of animal feed, anaerobic treatment, and landfilling [69]. Pectin is produced from by-products such as citrus peel or apple pomace. The processing includes pectin extraction with hot water acidification. It then undergoes filtrations and centrifugations and is finally precipitated with alcohol [70]. Research has been conducted on the valorisation of FW as adsorbents for dye and toxic constituents removal from contaminated waters via adsorption, which is a well-established technique. To highlight their significance in enhancing overall modeling performance and producing the best results, we integrated food waste-based adsorbents with communication and information adsorption isotherms, technology techniques, and kinetic models to find their usefulness [71]. Dairy processing processes, including chilling, pasteurization, and homogenization, generate a significant amount of water waste throughout the milk-processing process. These water pollutants contain significant amounts of dissolved sugars, proteins, milk products, possible residues of additives, and minerals. Fish heads, viscera, carcasses, and liquid waste, including cleaning and washing water output, blood-containing water from drained fish storage tanks, and brine, are produced by marine processing setups. A significant source of nutraceuticals, seafood, and its by-products accommodate bioactive substances. They contribute to human well-being and add nourishment to the diet [68]. Grain processing includes handling different kinds of grains, granules, bulk material, seeds as well, as vegetable oil and includes grading and cleaning, seed processing, drying, out loading and conveying, storage, filtration, control automation, aspiration, and vegetable oil processing. India delivers surplus sustenance grains of about 200 million tons per annum. According to the ministry of agriculture, sustenance grains achieved 270.10 MT in FY16. The oil extraction process produces an expansive amount of by-products in the form of oil cakes. In every phase of grain handling, biodegradable waste is created, which includes wastewater [6]. Pulses, cereals, and other grains are processed into by-products, which are rich sources of minerals and phytochemicals (bioactive substances), which are also beneficial for their beneficial technical and nutraceutical qualities. Brewer-wasted grains, lingering brewing yeast, and trub are referred to as wet brewery wastes, and water is widely used to clean, wash, and sanitize the brewery units [72]. The majority of the organic waste, such as spent malt and hops, is used as animal feed and for soil improvement [73]. Various by-products obtained from the food waste are summarized in Table 4.

## 5. Reutilisation of FW

### 5.1. Bio Processes-Solid State Fermentation (SFF)

In order to get energy, microorganisms use the process of anaerobic metabolism known as fermentation to break down organic substances. It is a low-waste process that uses little energy and costs little, and it may be used to turn organic waste into products with additional value [95]. In solid-state fermentation (SFF), yeasts, bacteria, and fungi grow on surface of different kinds of organic component, which provide physical support for their growth without the addition of water. The catabolism of proteins, lipids, and carbohydrates contributes to the production of primary metabolites, which are then converted into a mixture of aromatic compounds subsequently [96]. When by-products or agrifood wastes are exploited as a substrate to support the initial growth of microbes, glucose supplementation is necessary. However, high concentrations should be avoided as they can lead to catabolite repression phenomena. Mixing of different waste substrates can limit nutrient loss that occurs in SSF on agri FW. Keeping this aim, an SSF of mixed agrifood wastes with *Kluyveromyces marxianus, S. cerevisiae*, or an undefined mixed culture from kefir has been considered a promising idea for the development of biorefineries that aim to produce biomasses and volatile aroma compounds [97]. The production of specific compounds can be induced by the addition of precursors. For example, valine and leucine, when added to growth substrates such as agrifood wastes, isoamyl acetate is formed with a strong banana aroma due to the Ehrlich pathway, which catalyzes the amino acid and esters are produced as final products. The production of aroma compounds is a promising field for SSF application. The flavored compounds can be obtained directly from natural matrix, chemically synthesized, or derived from biotechnological processes including extraction. These compounds can be utilised in pharmaceutical industries, cosmetic industries, and chemical industries to improve or modify the original smell of the product. By doing this, the consumer market also accepts the products, and their importance enhances [96,98]. Many value-added products can be recovered from agrifood waste substrates after the process of fermentation, such as pigments, plastics, antibiotics, biosurfactants, hydrolytic enzymes, bioactive compounds, and pesticides, as mentioned in Table 5.

### 5.2. Mushroom Production

Mushrooms can be epigeous or hypogeous in origin and constitute a unique fruiting body. It is a protein-rich food and can be used in bioremediation. Mushroom production is an exquisite example of the regaining of food proteins by making use of biological processes in large or small-scale manner accordingly to lignocellulosic materials. It is a method of biotechnology for the process of valorization of agro-industrial waste. It also shows the strength of the economic as well as ecological points through the transformation of residues using microbes [107]. Researchers have reported 16 different agricultural-industrial wastes for growing *Pleurotus tuber-regium* whose varieties is commonly called oyster mushrooms. Application of these resulted in a variation to edible protein in relation to mushroom fruit bodies. Bahiagrass and banana stalks can also be utilized for the preparation of *Pleurotus sajor-caju* production [108]. The results for the production of it using Bahia grass and banana stalks as a substrate confirmed that no other replacements, viz rice bran or wheat bran, is required for the positive growing of the mushroom. In southern America, a study was conducted where coffee husks were used as a substrate for the cultivation of oyster mushrooms [109]. Scientists have also cultivated *Pleurotuseous* and *Pleurotus Platypus* by using agricolture-industrial unused. With paddy straw as a substrate found an increased amount of lipids, proteins, carbohydrates, etc. They even suggested that edible oyster mushrooms should be used for high protein content and a substrate of paddy straw for the successful production of mushrooms [110].

### 5.3. Anaerobic Co-Digestion (AcoD)

The digestion of two or more compounds that have complimentary properties exhibits improved performance and is anticipated to produce more biogas and have a better nutritional balance than mono digestion. Leachate from municipal solid waste (MSW) is a promising co-substrate for AcoD with FW because it has a high ammoniacal nitrogen concentration and tends to improve the ability to buffer, which can enhance the stable supply of nutrients for bacteria. AcoD of FW with MSW has also been accomplished at an industrial scale [111,112,113]. There are huge amounts of refractory organic matter in MSW leachate and FW, which can be converted to bioenergy through AcoD based integrated treatment process. It can not only provide known benefits, such as dilution, improving bioenergy production, and improving buffering capacity but also can promote refractory organics degradation and improve digestive dewaterability by using the proposed innovative integrated process, which is based on AcoD of FW. Mass balance analysis of the proposed process (volume ratio 1:1) depicts that the energy conversion efficiency can increase by 12% to 18% as compared to mono digestion; thus, AcoD could be a promising way to recover energy in the near future. However, it is important to choose an appropriate co-digestion proportion to promote the synergistic effect of AcoD cause of the huge difference in the properties of FW in various places, such as China especially, in terms of industrial applications [113,114]. Moreover, the dehydrated solid digestate from FW can be converted to biofertilizer after the treatment process such that the whole process reaches zero solid discharge. Studies focus on the environmental impact of using ionization radiation pre-treatment technologies in the process of anaerobic digestion of food waste and even compare it with that of traditional hydrothermal treatment. The introduction of ionization radiation pre-treatment causes the treatment of food waste via anaerobic digestion to have an environmental impact in 14 of the 18 categories that are analyzed as per the results, which is the lowest environmental impact. Pre-treatment is the most energy-consuming unit in food waste treatment and accounts for about 71–75% of the total energy consumption [115,116]. Liquid leachate was utilised as feedstock in another investigation to validate the effects of bio-electrochemical treatment under diverse circumstances. According to the findings, at an initial pH of 8.13 and a voltage of 0.7 V, the methane yield increased by 77.5% while the CO_2_ yield reduced by 16.0% when compared to the control without electrodes. Higher voltages of 1.1–2.0 V contributed to higher methane and hydrogen yields. Increased buffering capacity due to cathodic hydroxide production made the startup process more stable. The greatest methane output of 526.7 mL/gVS was observed with an immersed electrode surface area of 25.2 cm^2^/L and lower carbon dioxide and hydrogen concentrations [117].

### 5.4. 3D/4D Food Printing: Extrusion Technology

By enabling automated food customisation, on-demand food production, and reduced food waste, three-dimensional/four-dimensional printing, also known as additive manufacturing (AM), can leads to food value chains customer-desirable and stable [118]. AM reduces food waste by blending and combining low-value by-products like meat off-cuts with fresh fruits and vegetables to create a puree, which is then seasoned with spices and herbs. The puree is then 3-D printed, and after baking and dehydrating these prints, a sturdy and crispy product is produced. In a recent experiment, a 3-D printer based on extrusion (Controlled Additive-Manufacturing Robotic Kit) was used to create value-added functional cookies from grape pomace and broken wheat [119]. In another study, potato peel powder and whole wheat flour and are used to formulate instant noodles via extrusion printing technique with conditions optimized at 600-mm/min printing speed, 600-rpm extrusion motor speed, and 6-bar pressure for a 1.28-mm-diameter nozzle by CARK printer [120]. Fortified vegan protein was developed by AlgaVia, a company from the USA that used microalgae to develop a protein powder with impressive functional attributes such as being non-allergenic, gluten-free, and a good source of dietary fiber. By employing the proteins from wheat and soy, high-moisture extrusion is used to create meat analogs (MA). Comparative analyses of the MA and Chicken Breasts (CB) liberated peptides’ physical and chemical characteristics, in vitro digestion, and cellular absorption were performed; as a result, MA showed a advanced hardness but a decrease degree of texturization than the CB [121]. Another example of waste utilization is the edible insects that are dried into a powder; flour is mixed with icing butter, cream cheese, water, gelling agent, and flavoring to obtain the right consistency to go through the nozzle of the 3-D printer. Biologically active metabolites, flavouring compounds, and enzymes may be created from residues from the current food processing and agriculture industries as environmentally benign, sustainable raw materials for food printing [118].

### 5.5. Thermal Conversion and Composting of FW

Combustion convert carbonaceous solid waste into energy but has relatively lower efficiency and also cause air pollution. Between 400 and 1000 °C are used for pyrolysis; the byproducts include biochar (solid wastes), bio-oils (liquid), and other gaseous compounds. In comparison to combustion, gasification of food wastes produces hydrogen and biochar with lower greenhouse gas emissions; as a result, it is more efficient and environmentally friendly. Composting food waste is a slow process when compared to other reutilisation techniques, such as fermentation, anaerobic digestion, and thermochemical conversions. According to reports, a composting process can take anywhere between 3 and 8 months to complete unless it is sped up with the inclusion of worms, chemical activators, natural minerals, regular mixing, or other additions [122,123]. Aeration rate, carbon/nitrogen ratio, microbial succession, composition, moisture content, particle size, porosity, and temperature are the key parameters that impact the efficiency of food waste composting. Due to the high fat and moisture content of most food waste, composting requires special care. The anaerobic phases of composting, which produce methane (CH_4_), ammonia (NH_3_), nitrous oxide (N_2_O), and different greenhouse gas production, have a significant detrimental influence on the environment. In a nutshell, composting is not a desired alternative in food waste management [7].

## 6. Products Recovered from Industrial Wastes

**Figure 1:**
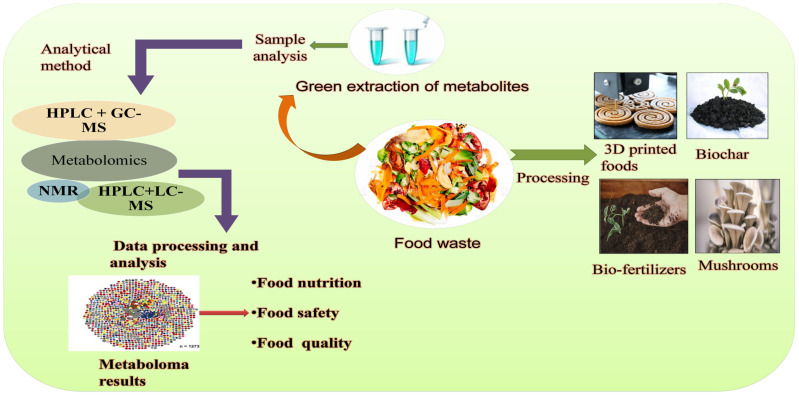
Schematic illustration of reutilisation of Food waste.

Biochemical components of FW have attracted the policymakers and researchers to recover various types of value-added products viz single cell protein, enzymes, bio-fertilisers, biochar and bioplastics, antioxidants, antibiotics, and biofuels and are discussed in this section (Figure 1).

### 6.1. Bioplastics and Biopolymers

Traditional petroleum-based plastics can be replaced with bioplastics for environmentally friendly food packaging [124]. Presently, edible films are being researched more and more to address the problem of microbial food spoilage, particularly by using plant extracts to create delicious materials for packaging. Natural polymer matrices infused with plant extracts are suitable for using casting techniques to create edible films. Chemical reaction with active compounds in plant extracts and the reactive groups in the polymer chain improved the structure and physicochemical properties of the film system [125]. FW is utilized to produce organic polymers, such as Polyhydroxybutyrate (PHB), Poly 4 hydroxybutyrate (P4HB), Polyhydroxyalkanoates (PHA), Polyhydroxyvalerate (PHV), Polyhydroxyoctanoate (PHO) and polyhydroxyhexanoate (PHH), and their copolymers (e.g., Poly-3-hydroxybutyrate (P3HB)), are the most commonly used bioplastics and are completely biodegradable into water and CO_2_ within a few months after burying [126]. Microbes, likse *E. coli*, *Clostridia Sp, Ralstoniaeutropha, Bacillus megaterium,* and natural isolates of *Azotobacter, Agrobacterium, Actinobacillus, Rhodobacter,* and *Sphaerotilius,* utilize food waste as substrate and produce polymers as either intracellular metabolite or as carbon reserves when nitrogen availability is limited and carbon is present in excess [127]. *Halomonas boliviensis* was grown by Pleissner et al. in bakery waste hydrolysate, saltwater, and PHB that made up about 30% of the dry cell weight and accumulated intracellularly [128]. Biopolymers obtained are the natural macromolecules produced by living organisms, and they are the repeating units of aliphatic polyesters such as sugars, amino acids, and fermentative products from agricultural wastes, which contributes to their functionality [129]. When sprayed directly on the ground, biopolymers derived from pectin present in apple and orange peels, alginates from algae, and chitosan found in crustacean shells treated with waste vinegar were successfully trialled in the replacement of films for agricultural soil mulching; they are used as weedicide film and, once its protective function is performed, become excellent plant fertilisers. They were also utilised in food packing in gel form [130].

### 6.2. Biofilms and Probiotics

Edible films operate as a barrier amongst the external environment and the food component, preventing direct interaction of the food with atmosphere and bacteria, lowering the rate of respiration and thereby increasing shelf life. However, the local fruits and vegetable vendors are not able to buy them because of their high cost. A cost-effective method for vegetable and fruit vendors has developed the solution for dip-coating and nanofibre coating using a blend of silk fibroin, Poly Vinyl Alcohol (PVA), curcumin, and honey by utilizing the techniques such as electrospinning and dip-coating [131]. By adding biochar that has been connected to biofilm made from food waste, researchers have developed a potential method to remove heavy metals from wastewater. Lead (Pb) and cadmium (Cd) adsorption efficiency was improved when the biochar was modified by bacterial biofilm, which decreased the specific surface area but increased the average pore size [132]. In a different research, a gravity sewer system was set up to examine how the addition of FW affected biofilm growth, the population of sulfate-reducing bacteria (SRB), and the possibility for sulphide generation. Long-term FW addition altered the sewage biofilm’s properties, resulting in an increase in thickness (by 32%), dry density (by 13%), and extracellular polymeric compounds (by 141%). The findings showed that adding FW to a sewage biofilm can encourage SRB development and sulphide production [133]. In this study, discarded fish scale-derived gelatin, chitosan, and CaCO_3_ nanoparticles were combined to create an eco-friendly edible film for post-harvest fruit (Longan and banana). The nanocomposite film displayed multifunctional capabilities such as UV absorption, oxygen screening, antibacterial activity, non-toxic nature, and mechanical properties and can be easily wiped off from fruits before consumption. The results revealed an effective increase in the shelf life of longan by more than three days and banana by more than five days [134]. Cheese whey-derived products, especially lactoferrin showing anti-microbial activities, can be incorporated with an edible film that can be made with a base component with plasticizer additives to make flexible and increase the shelf-life of perishable food products [135]. In a study conducted by Yin et al. (2013), Probiotic microorganisms such as one strain of *Lactobacillus*, two strains of *Bacillus*, and two strains of yeast, were selected and mixed at the same ratio and cultured using the kitchen waste as a culture medium at a pH of 7.2 and temperature of 37 °C. After 24 hours, the total viable cell count reached 2.24x10^10^ CFU/g, exceeding the level attained in pure cultures of any individual probiotic strain. As a result, occurrence of yeasts and *Bacillus* species facilitated the growth of the *Lactobacillus* strain [136].

### 6.3. Single-Cell Protein (SCP)

SCP, which is derived from bacteria, yeast, moulds, and algae, is a significant alternative to animal, fish, and human protein. SCPs are made from a variety of FW, including cleaning citrus wastes, sugarcane bagasse, soy molasses and yam peels [10]. The composition of the substrate and the medium affects how much SCP is formed. From beetroot pulp, mixed cultures of *Kluyveromyces marxianus* and *Trichoderma reesei* produced about 51% of SCP [137]. The protein constitutes all amino acids as per the guidelines given by FAO. Mondal et al. reported the production of SCP from the waste part of the fruit by using orange and cucumber peels as the substrate for the manufacture of SCP via submerged fermentation of *S. cerevisiae*. They concluded that cucumber peels produced more amount of protein when compared with orange peels [138]. Therefore, recommendations that these fruit wastes could be converted into SCP by using appropriate microorganisms. When agro-industrial waste is released into the environment, phenolic compounds and other harmful substances it contains may worsen the ecosystem. Fruit waste thrown in landfills slowly rots there and emits methane, a powerful greenhouse gas that absorbs 21 times more heat than carbon dioxide in the atmosphere. Fruit peel recycling or reuse is so urgently needed. In addition to meeting the demand for protein around the world, using agro-waste in SCP production can reduce environmental degradation brought on by waste disposal. These agro-wastes are made up of sugar, starch, and other cellulose components that can be metabolised by microorganisms through the production of extracellular enzymes. They are utilised as a substrate for the chosen microorganisms. Cellulose, hemicellulose, and lignin make up the majority of the components of lignocellulosic wastes, such as agricultural residues and fruit peels. Sugars are produced from cellulose, usually through the action of acids or cellulolytic enzymes. Enzymes from malt or molds hydrolyze starch resources, such as leftovers from root crops, including potatoes, corn, and cassava, into fermentable sugars. Like pineapple waste extract, cane, molasses, and fruit waste extract include beneficial ingredients, primarily sucrose, fructose, glucose, and other nutrients. The products produced from the biological conversion of agriculture -industries wastes are inexpensive, possess high protein [10,139].

### 6.4. Bio Fertilisers, Biochar, and Biofuels

Composting is deals with promoting aerobic biological breakdown of the organic solid portion, which greatly lowers the amount of trash landfilled. It is the simplest and most often used method for quickly stabilising solid organic components in aerobic setup. Aerobic microorganisms play a key part in the change of solid organic components into compost, carbon dioxide, and heat in this course [140]. The effectiveness of the composting procedure is enabled by the parameters such as the different populations of microbes, moisture content, aeration, temperature, biodegradable portion, and pH [10]. In certain circumstances, a specific microorganism is introduced to aid expedite the process of composting. When related to the production of synthetic fertilisers, compost saves a significant amount of water and energy. According to Chang and Chen, the water-carrying ability of the composting feedstock is the key physical attribute that has a detrimental influence on the composting rate [141,142]. In most cases, this procedure occur under stringent anaerobic conditions, due to which incomplete combustion of feedstock will produces biochar, also known as black carbon [143,144]. It consists of huge amounts of carbon, low-density material, and is quite porous. The alignment of the substrate and decomposition temperature has an important impact on the physicochemical features of the produced biochar. Biochar a high potential to avoid the emission of greenhouse gas, i.e., about 870 kg of carbon dioxide per ton of substrate used for the process of pyrolysis. It is an serve to remove organic and inorganic compounds from wastewater. It has a high holding capacity, hence, is used as a soil conditioner to enhance soil fertility and nutrient content for promoting the growth of plants and crop residues [145]. It is not readily decomposed and retained in the soil for longer and has several advantages such as (1) nutrient leaching, (2) total nitrogen, (3) reducing the level of biological oxygen demand, (4) fewer fertility requirements, (5) increased soil fertility, (6) soil water management, (7) increased soil fertility, and (8) increased plant development [146]. Biomethane, Biohydrogen, Biohytane, and biodiesel are some examples of biofuels produced by anaerobic co-digestion or fermentation of FW [137].

### 6.5. Organic Acids and Enzymes

Through the valorization of FW to different value-added products, the rate-limiting step is defiance down complex carbohydrates in simple sugars. FW contains large amounts of carbohydrates, and with the help of scarifying enzymes, carbohydrates are hydrolyzed to monosaccharides [63,147]. Enzymes, such as lipase, protease, pectinase, amylases, and cellulase, are obtained from various types of FW via SSF. Outer layer(peel) of potato is fermented by *Bacillus* at 35 °C; 7.5 pH to give around 676 U/mL of amylase. Microbes viz *Penicillium Sp., Aspergillus Sp.,* and *Bacillus Sp*. can ferment multiple FW, such as orange peel, apple pomace, and citrus wastes, and manufacture pectinase. *Pseudomonas aeruginosa* acts upon shrimps to yield about 15,000U/mL of enzyme protease within 24 h as the waste was fermented at 37° Celsius [10]. Alkan et al. at pH 7 and 37 °C stated that various waste products viz as melon wastes, banana and watermelon wastes were fermented by *Bacillus* coagulants to yield a titer value of about 148.9 U/g of lipase extracted from melon waste. Petrochemicals can be replaced by bio-based platform chemicals, including fatty acid, lactic acid, succinic acid, tartaric acid, citric acid, synthesis, 3-hydroxypropionic acid, 1,3-propanediol, and 2,3-butanediol. Because food waste contains fermentable proteins, carbohydrates, and lipids, it is thought to be the optimum feedstock for making these platform chemicals [102]. Organic acids, like as acetic acid, fumaric acid, citric acid, gluconic acid, and propionic acids, are also produced via valorization of FW [95].

### 6.6. Antibiotics and Biocontrol Agents (BCAs)

Different agricultural wastes are utilized for different kinds of microbes productions that kill or selectively inhibit other microbes at very low concentrations. Researchers have used corn cobs, rice hulls, and sawdust as raw materials for the preparation of oxytetracycline [59]. Asagbra et al. prepared oxytetracycline using ground nut shells as raw material with a strain of *Streptomyces rimosus* through SSF [148]. Vastrad and Neelagund reported the preparation of extracellular *rifamycin B* with the help of *Amycolatopsis* and Mediterranean MTCC 14 with oil-pressed cake, an agricultural waste by SSF. Agro-industrial wastes such as ground nut shells and coconut oil cake showed maximum antibiotic production [59]. Kitchen waste, fruit and vegetable waste, sugarcane baggase, winery waste, and wastewater treatment plants waste are used as substrates for the mass production of BCAs such as Trichoderma sp., Pseudomonas sp., and Bacillus thuringiensis (Bt) to achieve the goals of the Integrated Pest Management Program (IPMP) [149]. Due to high reliance on antibiotics, bacterial resistance has developed across the human food chain, and the efficiency of antibacterial medicines has also decreased. As a result, one of the research areas with the highest rate of growth is the use of phages as effective biocontrol agents in food production industry [150].

### 6.7. Food Additives and Antioxidants

Food additives are utilised for their diverse functions, such as reducing food perishability, microbial degradation, coloring or flavoring agents in foods, ensuring safety and improved characteristics, and ensuring greater food variability for the population. Vidhyalakshmi et al. (2012) reported xanthan, which is exopolysaccharide prepared from *Xanthomonas* species, as a food additive and is a cost-effective product produced from different agricultural-industries waste residues as they produced xanthan with the help of *X. citri, X. campestries, X musacearum,* and *X. oryzae* by SSF [151]. The highest xanthan was produced by *X. citri,* i.e., 2.90 g/50 g, followed by 2.87 g/50 g, 1.50 g/50 g, and 0.50 g/50 g by *X. musacearum, X.campestries, and X. oryzae,* respectively [59]. Food industry wastes, such as seeds and peels, rich in antioxidants. Barba et al. green strategies for extraction of antioxidant bioactive compounds from winery wastes and by-products such as grape stalks, grape marc, and grapes seeds. Grape seeds and green tea serve as rich sources of antioxidants, vitamin E, and flavnoids [152,153]. (Another research conducted by Amado et al. depicted antioxidant extraction from potato peel waste). The optimal extraction conditions for phenolics and flavonoids were 34 minutes of extraction time, 89.9 °C, and ethanol concentrations of 71.2% and 38.6%, respectively. In a summary, the study discovered potato peel to be a strong source of antioxidants that may effectively minimise oil oxidation [154].

## 7. Challenges Faced during Reutilisation of FW

FW quantification is currently inadequate in the food supply chain, due to restricted data on its quality and homogeneity, as well as variances in national waste legislation implementation. Regardless of the availability of several traditional techniques of landfilling or biogas production used to harness FW, its heterogeneous character and high moisture content continuously impede successful waste conversion. Food packaging adds to the severity of the situation as it predominantly relies on plastics, which contributes to micro-plastic pollution [155]. There is uncertainty in adequate food waste collection, storage facilities, cooking methods, cultural lifestyles, and bioconversion of FW to useful by-products is cited as a significant barrier in proper food waste management. Natural disasters, pandemics, and civil instability may all contribute to food loss and waste. The priority under such conditions is to stay alive and safe, and there is a shift away from harvesting and food production or processing. has an impact on the wholesomeness and integrity of such food items directly from the farm, even before they reach merchants and consumers. Many items are left unattended with minimum monitoring, even before they reach merchants and customers [156].

## 8. Future Perspectives and Conclusions

Food waste-related matters in developing countries are currently a prominent threatening factor for sustainable development. This proposed article has deeply investigated food waste reutilization techniques and innovations to tackle the problem. Studies of quantifying and efficient use of food waste vary significantly throughout the literature. Food waste studies with the inclusion of characterized food waste data, optimization of by-product methods, and application of technologies that already exist and their modification are crucial for the advancement of food wastage reduction. Integrated bio-refinery of food waste into a wide variety of value-added products is quite promising. Meanwhile, commodity chemicals derived from biofuels, biogas, biochar, and other chemicals help meet the global demand for large-scale reutilization of energy and resources. Fruits and vegetables act as adsorbents in addition to their usage in pharmaceutical and nutraceutical industries. The aim is to narrow down the path for evaluating the most technologically economic as well as efficient waste management technique for fruit and vegetable valorization in the longer run. There are a number of hurdles that prevent the conversion of food waste into useful value-added products. Several countries are handling this issue of rising FW. Without the acceptance of consumers of waste reduction approaches, no sustainable, eco-friendly way of management can succeed. Through education, consumers will see the value of these waste-derived products and their positive consequences on the environment. This eventually influences consumer behavior and promotes the purchasing patterns of food waste-derived value-added products in the market. Moreover, in recent times, consumers have become more conscious of energy expenditure; henceforth, they have been encouraged to reduce their home energy consumption by using other alternative strategies that aim at improving domestic energy efficiency. However, they do not compromise on the quality of the product or its performance. Preventing food waste in the food processing and agricultural sector requires improved technological solutions and infrastructure in harvesting, transport, distribution, storage, investment, and policies formulated by the government. The world’s population has been expanding rapidly with decreasing trend of natural resources at the same time, which raises concerns over food security at a global level due to the disparity between food poverty and food wastage.

## Figures and Tables

**Table 1:** Components of Pre-harvest agricultural residues.

Agricultural Residues	Chemical Composition (%*w/w*)						References
	Cellulose	Hemicellulose	Total Solids (%)	Moisture (%)	Lignin	Ash (%)	
Barley straw	33.8	21.9	ND	ND	13.8	11	[6]
Wheat straw	32.9	24.0	95.6	7	8.9	6.7	[6]
Cotton stalks	58.5	14.4	ND	ND	21.5	9.98	[6]
Sugarcane Bagasse	30.2	56.7	91.66	4.8	13.4	1.9	[13]
Sugar beet waste	26.3	18.5	87.5	12.4	2.5	4.8	[13]
Rice straw	39.2	23.5	98.62	6.58	36.1	12.4	[13]
Corn stalks	61.2	19.3	97.78	6.40	6.9	10.8	[13]
Sawdust	45.1	28.1	98.54	1.12	24.2	1.2	[13]
Oat straw	39.4	27.1	ND	ND	17.5	8	[13]
Soya stalks	34.5	24.8	ND	11.84	19.8	10.39	[14]
Sunflower stalks	42.1	29.7	ND	ND	13.4	11.7	[14]
Nut shells	25–30	25–30	ND	5.02–7.79	30–40	ND	[15]
Rice husk	21.5	33.1	89.41	10–15	14.6	20	[16]
Sugarcane straw	40.8	30.8	ND	35–50	25.5	5.3	[17]
Sorghum bagasse	40.4	35.5	ND	8.52	3.9	5	[18]

**Table 2:** Chemical composition of post-harvest agricultural residues/ industrial waste.

Type of Food Waste	Chemical Composition/Elemental Composition (%*w/w*)								
Waste	Carbon	Cellulose	Nitrogen	Hydrogen	Hemicellulose	Lignin	Ash	Moisture	References
Potato peel	1.3	2.2	9.1	ND	ND	20	7.7	9.89	[19]
Orange peel	3.87	9.21	ND	ND	10.5	0.84	3.5	11.86	[20]
Coffee skin	ND	23.77	ND	ND	16.68	28.58	5.36	ND	[21]
Pineapple peel	40.8	18.11	0.99	ND	47.72	1.37	ND	91	[22]
Wheat bran	49.81	24	0.7	6.11	ND	6	5.5	5.5–9.25	[23]
Rice bran	48.39	35	0.89	5.43	25	20	17	5.4	[24]
Corn cob	53.61	15	1.91	8.97	ND	6.2	ND	24-28	[25]
Corn stover	44	ND	0.8	6.3	17	ND	6.6	ND	[26]

**Table 3:** Summary of start-ups deploying AI and ML to manage FW.

Start-Up Name	Headquarters	Launching Year	Features	References
Winnow	London, UK	2019	➢ The AI system takes images of different items that are thrown away, runs the data through an algorithm, and then determines the financial and environmental consequences of lost food.	[64]
Wasteless	Tel Aviv, Israel	2013	➢ A premier real-time tracking to give customers dynamic pricing on the product expiration date in grocery retailers using machine learning. Save almost USD 1 billion per week.	[65]
Gamaya	Switzerland	2008	➢ Uses crop intelligence tools to help farmers increase crop production efficiency and decrease crop wastage.Utilizes drones with hyperspectral cameras to monitor pests, crop yields, water levels, and fertilizers.	[66]
Greyparrot	London, UK	2012	➢ Aims to advance the circular economy and determine the financial value of food waste. Sort and assist in recovery from massive waste using AI-based trash recognition technology.	[62]
The 77 Lab	MIT, Cambridge, USA	2022	➢ To boost overall production and improves the yield per hectare and reduces crop wastage, use of cutting-edge sensor and robotic technologies are employed. These eliminate weeding and concentrate on the production of sensitive crops, fruit, and sugarcane cultivation. Smartbots eliminate the problem of inefficient human farm work and save time by selecting the best produce from the crop plant.	[67]

**Table 4:** Summary of various by-products obtained from FW of different segments of the industry, along with their extraction parameters and benefits.

By-Product	Food Waste	Operation/Extraction Parameters	Use/Benefits	References
Fruit juice processing				
Pectin	Orange peel; Apple pomace	Extraction of pectin with hot water acidification, filtrations, centrifugations, and then precipitation with alcohol	Fat/sugar replacer, reduce blood cholesterol levels, prevents gastrointestinal disorders	[70]
Natural sweeteners	Fruit pomace	Chicory processing: liquid has evaporated, and the sugars were crystallized and dried	Lowers blood pressure, prevents heart disease and risks of diabetes, acts as anti-inflammatory substance	[74]
Low-calorie jam	Tomato pomace	Basic jam formulation with TSS value of 48°Brix	Decreases blood pressure, constipation, and risk of heart attack	[75]
Pectin as Corrosion inhibitors	Tomato peel	Extraction of pectin with oxalic acid/ammonium oxalate under reflux	Decrease in waste disposal of canning factory 71% corrosion inhibition efficiency	[76]
Antioxidants	Fruit pomace (beet root)	Extraction using organic solvents (ethanol, acetone, hexane) followed by fractionation and purification	Reduce risk of cancer and other diseases	[77]
Antioxidants	Olive fruit waste	Time-40 min; Temperature-55 °C; pH-5.75.	Better economic enhancement of antioxidant properties in fatty food than synthetic additives	[78]
Activated carbon absorbent	Grape waste	Impregnation ratio—6:1; activation temperature—600 °C; and activation time—60 min	Effectively removes cationic and anionic dyes from aqueous solution	[79]
Essential oil	Fruit pomace; berry seeds	Supercritical fluid extraction, cold pressing, and distillation	Antiseptic and antibiotic properties, natural decongestant, anti-inflammatory properties	[80]
Fibers	Fruit pomace	Grinding and centrifugation or mechanical dewatering until 1% dry matter	Reduce cholesterol, maintain blood sugar level, slow down fat absorption	[81]
Vegetable processing				
Potato fiber	Potato peel	Washing and drying in an oven at 60 °C/ 12 h, milling to a particle size of 500µm, sieving, and storage under refrigeration	Prevention of diseases	[82]
Carrot-based condensed milk	Carrot pomace	Vacuum drying	Abundant source of carotenoids, fibers, and phenolic compounds	[83]
Green pea powder (snack crackers and dry soup)	Green pea peels	Drying in an oven at 60 °C/12 h; grinding and sieving (500–600 µm)	Rich source of cellulose	[84]
Yogurt	Carrot pomace	Extraction of carotenoids by electrostatic extrusion; carrot waste beads concentrations of 2.5 and 5 g/100 g were added to yogurt.	Fortified yogurt with enhanced antioxidant properties	[85]
Dairy industry				
Whey powder	Whey	Spray drying	Animal feed, ethanol production	[86]
Lactic ferments	Skimmed milk, whey	ND	Fermentation process	[86]
Demineralised whey powder	Whey	Ion-exchange, elect-dialysis	Food products for lactose-intolerant people	[86]
Lactose	Whey	Crystallization of concentrated whey	Syrups	[86]
Whey protein concentrate	Whey	ND	Food and pharmaceutical ingredients	[86]
Whey cheese	Whey	Condensation	Ricotta cheese or messoer	[87]
Marine industry				
Edible gelatin	Fish and rejected parts	Extraction in hot water and air-drying	Stabilizers, Clarifiers, texturing agents, and preservatives used in medication formulation and dietary supplements	[88]
Industrial gelatin	Fish and rejected parts	Extraction in hot water and air-drying	Dyes micro-encapsulation	[89]
Collagen	Fish skins and bones	Cold-water extraction	ND	[90]
Bioactive	Fish rejected parts	Enzymatic hydrolysis of crustacean and mollusk marine waste	Anti-microbial, anticoagulant, antidiabetic, antihypertensive, hypo-cholesteraemic, and anticancer agents as promising nutraceuticals	[91]
Fish protein concentrate (FPC)	Fish and rejected parts	Solvent extraction	Limited applications due to legislative issues	[90]
Photographic	Fish and rejected parts	Extraction with hot water and air-drying	Electronics industry coating that is light-sensitive	[91]
Glue	Fish and rejected parts	Extraction with hot water and air-drying	Adhesive applications	[91]
Meat industry				
Blood	Animal carcass	Blood is approved for food use when it has been removed by bleeding an animal that has been inspected	Non-food substances viz fertilizer, feedstuffs, and binders; high levels of protein and heme iron as a result used in making blood sausages, blood pudding, biscuits, and bread; pharmacological use	[92]
Hides and skin	Animal carcass	ND	Shelters, clothing, finished products obtained from hides of pigs and cattle and sheep pelts includes leather bags and shoes, rawhide, cosmetic products, edible gelatine, sausage skins, and glue	[93]
Bone	Animal skeleton	ND	Marrow inside some of the bones can also be used as food, making soup and gelatine.	[94]
Glands and organs	Animal body	ND	Human foods comprise heart, brain, liver, lungs, kidneys, etc; stomach and uterus of pigs, the rumen, reticulum, absomasum and omasum of cattle and sheep, and thymus and testes of sheep and pigs	[94]
Brewery industry				
Animal feed	Brewers spent gain (BSG), hot trub	Formed during mashing process and removed before the boiling step of the brewing process.	Showed positive influence on the production efficiency in cattle, without affecting fertility; improvement in the milk yield and composition	[74]
Essential oil (myrcene, *α*-humulene, and β-caryophyllene)	Spent hops	Hydro distillation.	More affordable and environmentally friendly alternative to chemical insecticides; can be utilized for prevention of stored food-stuff	[74]
Food additives	BSG	ND	Enhancement of aroma binding properties and positive effect on gelling and emulsifying potential	[74]
Antioxidants	Brewers spent yeast (BSY)	Recovered by sedimentation before full maturation of beer at the final stage of the second fermentation and maturation	Lower the risk of development of certain diseases-cancer, cardiovascular and neurodegenerative diseases	[74]

ND-Not defined.

**Table 5:** Summary of value-added products obtained from food waste, including operations, experimental conditions, and product yield.

Value Added Product	Food Waste	Operation	Experimental Conditions	Product Yield	References
Bio plastic-Polyhydroxyalkanoates (PHA)	Organic fraction of municipal solid waste (OFMSW)	Fermentation in sequencing batch reactor	Volume—100 L; Temperature—25–28 °C; Cycle Length—0.25 days	76 g PHA/kg	[99]
Hydrogen gas- an alternative to fossil fuel	Food waste in China	Steam reforming process’s stoichiometric chemical equations with water–gas shift reaction	ND	221.13 × 10^9^ kg	[100]
Succinic acid	Bread waste	Solid-state fermentation in bioreactor	Volume—2.5 L; Substrate to inoculum Temperature—37 °C; pH—6.6–6.8; Time—48 h	47.3 g/L	[101]
Lipase enzyme	Melon waste	Solid-state fermentation in Erlenmeyer flask	Volume—250 mL; Temperature—37 °C; Time—24 h; pH—7.0	148 U/g	[102]
α-amylase enzyme	Potato peel	Solid-state fermentation in Erlenmeyer flask	Volume—500 mL; Time—24 h; pH—7.5; Temperature—35 °C	676 U/mL	[103]
Butanol and hydrogen	Food waste-moisture with known carbohydrate, protein, total fat ash content	Fermentation without enzymatic pre-treatment using amylolytic *Clostridium sp.* strain BOH3.	ND	ND	[104]
Ethanol and lactic acid	Meat, noodle, potato, and vegetable waste	Fermentation of food waste using indigenous consortium with *Saccharomyces cerevisiae*	ND	ND	[105]
Biochar	Food waste	Pyrolysis in Muffle furnace	Time—45–60 min; Heating rate—10 °C/min; Temperature—500 °C	71%	[106]

## Data Availability

Not applicable.
